# The Relationship Between Short-Term Mean Arterial Pressure Variability and Mortality in Critically Ill Patients

**DOI:** 10.3389/fcvm.2022.870711

**Published:** 2022-04-29

**Authors:** Chenwei Hou, Xin Wang, Yakun Li, Feilong Hei

**Affiliations:** ^1^Department of Cardiopulmonary Bypass, Fuwai Hospital, National Center for Cardiovascular Disease, Chinese Academy of Medical Sciences and Peking Union Medical College, Beijing, China; ^2^Netbrain Technologies Inc., Beijing, China; ^3^Center for Cardiac Intensive Care, Beijing Anzhen Hospital, Capital Medical University, Beijing, China

**Keywords:** mortality, blood pressure variability (BPV), mean arterial pressure (MAP), intensive care unit, average real variability

## Abstract

**Background:**

Increased or decreased blood pressure variability may affect the perfusion of tissues and organs, leading to acute kidney injury and death. This study was conducted to explore the relationship between mean arterial pressure variability and short- and long-term mortality in critically ill patients.

**Methods:**

We used patient data from the MIMIC-III database for cohort study. According to the recorded mean arterial pressure during the first 24 h in the intensive care unit, we calculated each patient’s two variability parameters –coefficient of variation and average real variability. The primary outcome was in-hospital mortality and the secondary outcomes were 28-day mortality and 1-year mortality. We conducted smooth spline models to examine the possible nonlinear associations between blood pressure variability and mortality. According to the smoothing curve, we further developed a two-piecewise linear regression model to find out the threshold effect. Multivariable logistic regression or Cox proportional hazards model was used to evaluate the relationship. Kaplan–Meier survival analysis for 28-day and 1-year mortality was performed. Subgroup analysis explored the factors modifying the relationship between them.

**Results:**

A total of 12,867 patients were enrolled in the study, 1,320 in-hospital death, 1,399 28-day death, and 2,734 1-year death occurred. The smooth spline showed death risk was the lowest when average real variability was around 7.2 mmHg. After adjusting for covariates, logistic or Cox regression showed the highest MAP variability level was strongly associated with increased mortality in the hospital (odds ratio: 1.44; 95% CI, 1.21∼1.72), at 28 days (hazard ratio: 1.28; 95% CI, 1.1∼1.5), and at 1 year (hazard ratio: 1.27; 95% CI, 1.14∼1.42) compared with the second level of average real variability group. The survival curve plot showed patients with higher average real variability had a higher risk of 28-day and 1-year mortality. This relationship remained remarkable in patients with low or high Sequential Organ Failure Assessment scores in the sensitivity analysis. The two-piecewise linear regression model showed that lower ARV was a risk factor for 28-day (HR 0.72, 95% CI, 0.57∼0.91) and 1-year mortality (HR 0.81, 95% CI, 0.68∼0.96) when ARV was less than 7.2 mmHg, higher ARV was a risk factor for 28-day mortality (HR 1.1, 95% CI, 1.04∼1.17) and 1-year mortality (HR 1.07, 95% CI, 1.02∼1.12) when ARV was greater than 7.2 mmHg.

**Conclusion:**

Blood pressure variability predicts mortality in critically ill patients. Individuals with higher or lower mean arterial pressure average real variability during the first day in ICU may have an increased risk of death.

## Introduction

In clinical practice, blood pressure (BP) is routinely measured physiological variables. Under normal circumstances, blood pressure variability includes fluctuations between beats —— very short-term blood pressure variability (BPV), fluctuations over 24 h —— short-term BPV, daily fluctuations——medium-term BPV, and visits fluctuations——long-term BPV. Blood pressure variability may be a response to external stimuli and changes in daily life, aiming to maintain the so-called “steady state” of blood pressure (1).

However, the continuous increase or decrease of BPV not only reflects the damage of the cardiovascular homeostasis regulation mechanism but also causes potential pathological damage by affecting organ perfusion. The role of BPV for cardiovascular disease or death has been studied widely in recent years. Studies have shown that the effect of BPV independent of mean blood pressure is related to target organ damage (TOD) in heart, blood vessel, brain, and kidney (1–3), and the increase of intraoperative blood pressure fluctuation lead to the increased mortality (4, 5).

Hemodynamics in critically ill patients are often unstable, but there are few studies on the impact of blood pressure variability on prognosis in critically ill patients. Moreover, the current research on blood pressure variability lacks the corresponding safety threshold, which limits the clinical application of this index. This study intends to explore the relationship between mean arterial pressure (MAP) variability and prognosis in critically ill patients by analyzing the data in intensive care multiparameter intelligent monitoring database III (MIMIC-III). We present the following article in accordance with the STROBE reporting checklist.

## Materials and Methods

### Study Design

We collected the patients’ data from the MIMIC-III database and performed a retrospective cohort study. The database is maintained by Beth Israel Deaconess Medical Center (Boston, MA, United States) and the Massachusetts Institute of Technology (Cambridge, MA, United States) and contains the medical information of over 40,000 patients who were hospitalized in the intensive care unit (ICU) from 2001 to 2012 at Beth Israel Deaconess Medical Center, including demographic characteristics, vital signs, laboratory and radiology results, medications, comorbidities, nursing notes, physician discharge summaries, and survival outcomes (6). Any researcher who wants to use the database must complete the “protecting human subjects” training and then can obtain access to the data.

### Study Population

All patient information was obtained from the MIMIC-III database(version 1.4) (7). The inclusion criteria were (1) age ≥18 years when entering ICU, (2) first entering ICU. The exclusion criteria were (1) length of stay in ICU <1 day, (2) interval between two adjacent blood pressure monitoring on the first day of admission >1 h and blood pressure monitoring records on the first day <24 times, (3) lack of covariate data for multivariate adjustment. Except for blood pressure records, no other variables were missing.

Since the records of all patients in the MIMIC-III database were anonymous, the institutional review committee of Beth Israel Deaconess Medical Center waived the requirement for individual consent of patients.

### Data Extraction

The following patient data were extracted from the MIMIC-III database: age, gender, ethnicity, length of ICU stay, the first Sequential Organ Failure Assessment (SOFA) score, ventilation therapy (noninvasive and invasive), medication usage on day 1 of admission to the ICU, diseases (diabetes mellitus, hypertension, coronary heart disease, congestive heart failure, cancer, chronic lung disease, chronic liver disease, chronic kidney disease, sepsis), complete invasive mean arterial pressure(MAP) records, survival outcomes (in-hospital, 28-days, 1-year). Vasopressor medications included norepinephrine, phenylephrine, dopamine, isoproterenol, epinephrine, and vasopressin, and vasodepressor medications included nitroprusside, nicardipine, labetalol, esmolol, and diltiazem. Chronic lung disease includes obstructive chronic bronchitis and chronic obstructive asthma. Chronic liver disease includes a history of viral hepatitis, autoimmune hepatitis, non-alcohol fatty liver disease, cirrhosis, or liver failure as listed in the electronic health. All diseases in our study were classified using the International Classification of Diseases, Ninth Revision (ICD-9). Data were extracted using Navicat software.

### Blood Pressure Variability

Blood pressure variability(BPV) was assessed with 2 different indexes: coefficient of variation (CV), average real variability (ARV). Standard deviation (SD) is the simplest statistical measure for describing variation but is highly dependent on the individual’s BP level. CV is derived from the SD by dividing it by the mean. Consequently, CV is less influenced by BP level and is therefore considered an applicable index in variability studies (8, 9). ARV takes also into account the order of measurements and is calculated from the mean absolute difference between consecutive BP measurements (10). The ARV index and CV index are calculated using the following formulas (4):

$$A\,R\,V=\frac{1}{\Sigma \,w}\times \sum_{k=1}^{N-1}w\times \left|B\,P_{k+1}-B\,P_{k}\right|$$

$$C\,V=\sqrt{\frac{\sum_{k=1}^{N}{\left(B\,P_{k}-\bar{B\,P}\right)}^{2}}{\left(N-1\right)}}/\bar{B\,P}$$

where, *N* denotes the number of valid BP measurements in the data corresponding to a given subject, and *k* ranges from 1 to *N*-1. *W* means the interval time between two adjacent blood pressure records. In this study, we, therefore, used ARV as our main exposure variable because it can be relatively easily calculated in clinical practice, and a universal reference frame can be defined.

We discarded BP readings with a mean arterial pressure <25 or >250 mm Hg to retain physiologically meaningful readings in the analysis and to ensure the reliability of variability indexes. Baseline blood pressure was the average of MAP on the first day of ICU admission.

### Outcomes

The primary outcome measure in our study was hospital mortality. The secondary outcome measures was 28-day and 1-year mortality. In addition, in-hospital and 28-day mortality were defined as short-term mortality, and 1-year mortality was defined as long-term mortality.

### Statistics Analysis

Data for continuous and categorical variables were presented as median with interquartile range and frequency with percentage, respectively. Continuous and categorical variables were compared using the Mann–Whitney test and the X^2^ or Fisher exact test, respectively.

To assess the association of BPV (as a categorical or continuous variable) with the study outcomes, we used multivariate logistic regression models for in-hospital mortality and Cox proportional hazards models for 28-day and 1-year mortality. To assess confounding, we entered covariates into a Cox regression model in the basic model or eliminated the covariates in the complete model one by one and compared the regression coefficients. Those covariates altering initial regression coefficients by more than 10% were included such as age, sofa, average of MAP, coronary heart disease, chronic liver disease, sepsis. Considering that gender, hypertension, diabetes, use of vaso-drug and ventilation therapy in the first day are also important clinical covariates, they were also included in the final model. The odds ratios (ORs) were generated for logistic regression and hazard ratios (HRs) were generated for Cox proportional hazards, with their 95% CIs. We divided the population into 4 groups by quartiles of ARV. We estimated hazard ratios or odds ratios contrasting the risk for mortality in each level versus the average risk in the second level which the risk is lowest. Survival curves were constructed by the Kaplan–Meier method and compared by the log-rank test. We investigated the association between BPV and outcome in subgroups by SOFA score. We tested for interaction to determine whether the relative effect of BPV varied significantly among SOFA levels by introducing an interaction term in the models.

To explore the possibility of a non-linear relationship between BPV and outcomes, we fitted models with smooth spline and carried out nonlinear tests. We found that the risk of mortality was the lowest when MAP ARV is around 7∼8 mmHg. According to the smoothing curve, we further developed a two-piecewise linear regression model to find out the threshold effect, with adjustment for potential confounders. *P*-values <0.05 were considered statistically significant.

All analyses were performed using R Statistical Software (The R Foundation).^1^ Navicat software was used to extract population samples and variable information. Python was used to calculate the blood pressure variability index.

### Subgroup Analyses

The SOFA score as a measure of organ dysfunction is associated with patient outcomes. We conducted subgroup analyses to determine whether the results persisted even when the severity of the clinical status changed. Based on the median first SOFA score, all patients were divided into the low (<5) and high (≥5) SOFA score groups where 5 was the median of SOFA. Logistic regression and Cox proportional hazards models were used to evaluate the BPV and outcomes in the subgroups.

## Results

### Patient Selection, Characteristics, and Outcomes

**FIGURE 1:**
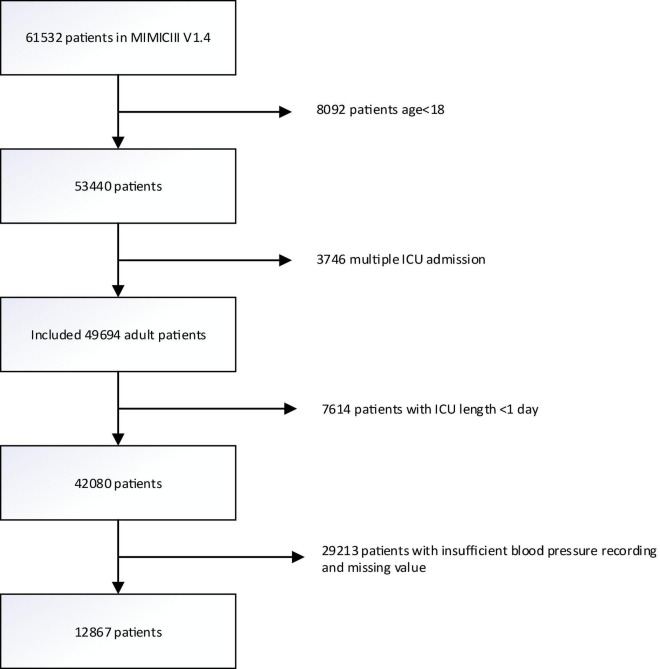
Flowchart of participant selection. A total of 12,867 patients were included in the analysis. MIMIC-III, Multiparameter Intelligent Monitoring in Intensive Care III.

A total of 61532 ICU records were screened from the MIMIC-III database. We excluded 3746 records with multiple admission to the ICU, 8092 records with age <18, 7416 records with the length of stay in ICU <1 day, and 29213 records with blood pressure recording interval greater than 1 h and recording times less than 24 in the first 24 h after admission to the ICU. Finally, 12867 patients were included in the analysis (Figure 1). Among them, 1320 in-hospital death, 1399 28-day death, and 2734 1-year death occurred. The median age of all patients was 65 years old, the median SOFA score was 5, and males accounted for 61.5%. Patients with higher ARV levels tended to be older and female, and to have a history of hypertension, diabetes mellitus as well as other comorbidities. There were also significant differences in outcomes. Table 1 shows the characteristics of the study population across quartiles of ARV.

**TABLE 1:** Characteristics of the study population grouped by the quartiles of MAP ARV.

Variables	Q1 < 5.7 (*n* = 3217)	Q2 ≥ 5.7 to < 7.2(*n* = 3216)	Q3 ≥ 7.2 to < 9.4(*n* = 3217)	Q4 ≥ 9.4 (*n* = 3217)	P
Female, n (%)	1111 (34.5)	1188 (36.9)	1233 (38.3)	1427 (44.4)	< 0.001
Age, Median (IQR)	63.0 (52.4, 73.1)	65.9 (55.6, 75.5)	68.4 (57.3, 77.5)	70.9 (59.2, 79.3)	< 0.001
**Ethnicity,n (%)**					< 0.001
1	2350 (73)	2316 (72)	2338 (72.7)	2237 (69.5)	
2	70 (2.2)	67 (2.1)	84 (2.6)	59 (1.8)	
3	164 (5.1)	164 (5.1)	177 (5.5)	229 (7.1)	
4	104 (3.2)	95 (3)	81 (2.5)	78 (2.4)	
5	529 (16.4)	574 (17.8)	537 (16.7)	614 (19.1)	
Mean MAP 1st day, Median (IQR)	73.7 (68.8, 80.0)	74.7 (70.2, 80.8)	76.0 (71.6, 81.8)	78.7 (73.5, 86.0)	< 0.001
SOFA, Median (IQR)	5.0 (3.0, 8.0)	5.0 (3.0, 7.0)	5.0 (3.0, 7.0)	5.0 (3.0, 7.0)	0.29
CHD, n (%)	1203 (37.4)	1395 (43.4)	1421 (44.2)	1224 (38)	< 0.001
Cancer, n (%)	577 (17.9)	540 (16.8)	606 (18.8)	602 (18.7)	0.123
CHF, n (%)	886 (27.5)	855 (26.6)	857 (26.6)	855 (26.6)	0.78
Chronic liver disease, n (%)	472 (14.7)	352 (10.9)	284 (8.8)	299 (9.3)	< 0.001
Chronic lung disease, n (%)	88 (2.7)	90 (2.8)	91 (2.8)	120 (3.7)	0.061
Chronic kidney disease, n (%)	185 (5.8)	195 (6.1)	188 (5.8)	183 (5.7)	0.925
Hypertension, n (%)	1395 (43.4)	1624 (50.5)	1657 (51.5)	1709 (53.1)	< 0.001
Diabetes mellitus, n (%)	833 (25.9)	921 (28.6)	958 (29.8)	921 (28.6)	0.005
Vaso-drug use 1st day, n (%)	1414 (44)	1450 (45.1)	1450 (45.1)	1486 (46.2)	0.354
Ventilation therapy, n (%)	2626 (81.6)	2722 (84.6)	2748 (85.4)	2758 (85.7)	< 0.001
Sepsis, n (%)	903 (28.1)	809 (25.2)	891 (27.7)	1058 (32.9)	< 0.001
LOS_hospital, Median (IQR)	8.7 (5.5, 14.5)	8.4 (5.8, 13.9)	8.8 (6.0, 14.5)	9.5 (6.2, 16.1)	< 0.001
death, n (%)	336 (10.4)	264 (8.2)	301 (9.4)	419 (13)	< 0.001
28-day death, n (%)	369 (11.5)	287 (8.9)	296 (9.2)	447 (13.9)	< 0.001
1-year death, n (%)	664 (20.6)	572 (17.8)	650 (20.2)	848 (26.4)	< 0.001

*Values are presented as median (inter-quartile range) or number (%). IQR, inter-quartile range; ARV, average real variability; MAP, mean arterial pressure; SOFA: Sequential Organ Failure Assessment; LOS, length of stay; Ethnicity: 1, WHITE; 2, ASIAN; 3, BLACK; 4, HISPANIC OR LATINO; 5, OTHER; CHD, coronary heart disease; CHF, congestive heart failure; LOS, length of stay.*

### Association Between Average Real Variability and Mortality

**FIGURE 2:**
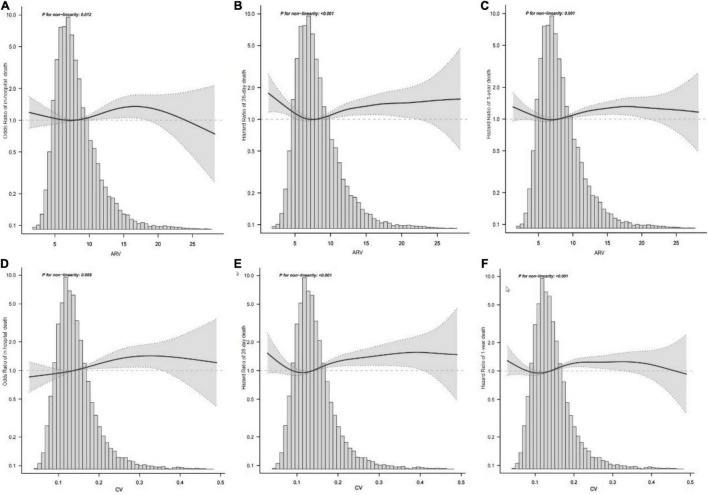
Association between MAP variability and mortality. Smooth spline plots of the association between MAP ARV and in-hospital mortality **(A)** or 28-day mortality **(B)** or 1-year mortality **(C)**, and the relationship between MAP CV and in-hospital mortality **(D)** or 28-day mortality **(E)** or 1-year mortality **(F)**. ARV, average real variability; CV, coefficient of variation; MAP, mean arterial pressure.

**FIGURE 3:**
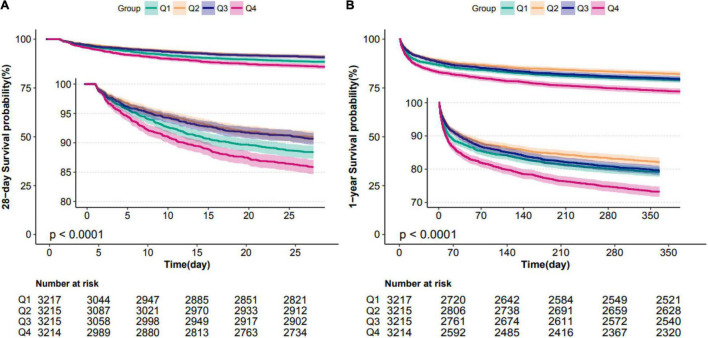
Kaplan–Meier survival analysis plots for 28-day and 1-year mortality with MAP ARV. The curves show that patients with higher MAP ARV in the ICU had lower rates of 28-day survival **(A)** and 1-year survival **(B)**. ICU indicates intensive care unit; ARV, average real variability; MAP, mean arterial pressure.

Patients in the fourth level of ARV group had higher in-hospital, 28-day, and 1-year mortality than other groups (13, 13.9, and 26.4%, respectively) (Table 1). To verify these findings, we used smooth splines to describe the relationship between BPV and outcomes (Figure 2). It was found that after adjusting the covariates, the relationship between ARV and outcomes showed a U-shaped curve, and the risk of mortality was the lowest when the ARV was about 7∼8 mmHg. The trend of the relationship between CV and outcome was similar (Figure 2). Multivariable analyses adjusted age, gender, sofa, average of MAP, coronary heart disease, chronic liver disease, sepsis, hypertension, diabetes, use of vaso-drug and ventilation therapy in the first day and showed that higher ARV levels were associated with increased risks of in-hospital mortality compared with the second group which the risk of death is lowest (adjusted OR 1.44, 95% CI 1.21∼1.72; *P* < 0.001), 28-day mortality (adjusted HR 1.28, 95% CI 1.1∼1.5; *P* = 0.001), and 1-year mortality (adjusted HR 1.27, 95% CI 1.14∼1.42; *P* < 0.001, Table 2).Kaplan–Meier survival curves revealed that the 28-day and 1-year probability of survival was higher in the fourth ARV group than the rest (Figure 3). According to the smoothing curve, we further developed a two-piecewise linear regression model to find out the threshold effect, with adjustment for potential confounders. The results showed that lower ARV was a risk factor for 28-day (HR 0.72, 95% CI, 0.57∼0.91) and 1-year mortality (HR 0.81, 95% CI, 0.68∼0.96) when ARV was less than 7.2 mmHg, higher ARV was a risk factor for 28-day mortality (HR 1.1, 95% CI, 1.04∼1.17) and 1-year mortality (HR 1.07, 95% CI, 1.02∼1.12) when ARV was greater than 7.2 mmHg. (Table 3).

**TABLE 2:** Multivariable Analysis of the Association between MAP Variability and Endpoints.

Outcomes	MAP ARV level categorical/continuous	Number of patients with event (%)	Adjusted OR/HR (95% CI)	Adjusted *P*-value
In-hospital mortality	Q2	260 (8.1)	Reference	−
	Q1	278 (8.6)	1.17 (0.97∼1.42)	0.105
	Q3	338 (10.5)	1.32 (1.1∼1.59)	0.003
	Q4	444 (13.8)	1.44 (1.21∼1.72)	< 0.001
	MAP ARV(per SD)	—	1.05 (0.99∼1.11)	0.117
28-day mortality	Q2	287 (8.9)	Reference	−
	Q1	296 (9.2)	1.22 (1.05∼1.43)	0.011
	Q3	341 (10.6)	0.95 (0.81∼1.12)	0.559
	Q4	475 (14.8)	1.28 (1.1∼1.5)	0.001
	MAP ARV(per SD)	—	1.05 (1∼1.1)	0.051
1-year mortality	Q2	597 (18.6)	Reference	−
	Q1	587 (18.2)	1.15 (1.03∼1.29)	0.015
	Q3	674 (21)	1.06 (0.95∼1.19)	0.319
	Q4	876 (27.2)	1.27 (1.14∼1.42)	< 0.001
	MAP ARV(per SD)	—	1.06 (1.02∼1.09)	0.002

*ARV, average real variability; MAP, mean arterial pressure.*

**TABLE 3:** Risk of death per 1 SD increase in blood pressure variability.

Outcomes	ARV < 7.2 mmHg		ARV ≥ 7.2 mmHg	
	OR/HR(95%CI)	*P*-value	OR/HR(95%CI)	*P*-value
In-hospital mortality	0.75 (0.57∼1)	0.054	1.05 (0.97∼1.13)	0.238
28-day mortality	0.72 (0.57∼0.91)	0.006	1.1 (1.04∼1.17)	0.001
1-year mortality	0.81 (0.68∼0.96)	0.015	1.07 (1.02∼1.12)	0.003

*ARV, average real variability.*

### Subgroup Analysis

When stratified by SOFA score (≥5 5898 patients versus <5 6969 patients), we found that our results almost persisted in the different subgroups after adjustment for all covariates. That is, the highest ARV group had a higher risk of mortality. In the group with low SOFA score, in-hospital, 28-day, 1-year mortality had ORs of 1.47 (95% CI, 1.06∼2.04), HRs of 1.49 (95% CI, 1.12∼1.99) and 1.24 (95% CI, 1.03∼1.49), respectively. In the group with high SOFA score, in-hospital, 28-day, 1-year mortality had ORs of 1.28 (95% CI, 1.03∼1.59), HRs of 1.18 (95% CI, 0.98∼1.41) and 1.26 (95% CI, 1.1∼1.44), respectively (Table 4).

**TABLE 4:** OR/HR With 95% CIs for mortality associated with MAP ARV in patients with different SOFA scores.

Group	Mortality		
	Hospital, OR(95%CI)	28-day, HR(95%CI)	1-year, HR(95%CI)
**SOFA < 5 (*n* = 5898)**			
Q2	Reference	Reference	Reference
Q1	1.11 (0.76∼1.62)	1.26 (0.91∼1.75)	1.1 (0.89∼1.35)
Q3	1.07 (0.75∼1.54)	1.03 (0.75∼1.42)	1.01 (0.83∼1.23)
Q4	1.47 (1.06∼2.04)*	1.49 (1.12∼1.99)*	1.24 (1.03∼1.49)*
**SOFA ≥ 5 (*n* = 6969)**			
Q2	Reference	Reference	Reference
Q1	1.21 (0.97∼1.5)	1.17 (0.98∼1.39)	1.14 (1∼1.31)
Q3	1.09 (0.87∼1.35)	0.94 (0.78∼1.13)	1.09 (0.95∼1.25)
Q4	1.28 (1.03∼1.59)*	1.18 (0.98∼1.41)	1.26 (1.1∼1.44)**

*, P < 0.05; **, P < 0.001; ARV, average real variability; MAP, mean arterial pressure; SOFA, Sequential Organ Failure Assessment.

## Discussion

In this study, we used ARV and CV to describe blood pressure variability. The results confirmed that there was a significant correlation between the variability of mean arterial pressure in the first 24 h after admission to ICU and in-hospital mortality, 28-day mortality, and 1-year mortality, independent of the confounding factors including BP levels. The relationships between BPV and outcomes were non-liner. In subgroup analysis, the results remained stable in populations with different sofa scores.

In recent years, many studies have explored the relationship between blood pressure variability and adverse outcomes. The traditional view is that the blood pressure threshold is a risk factor for in-hospital mortality or poor long-term prognosis. With the development of technology and concept, people are not satisfied with using only static indicators to describe the state of patients but prefer to use dynamic indicators to measure the general situation of patients. In the context of cardiac surgery, many models and scoring systems used to predict postoperative adverse outcomes, such as STS, EuroSCORE, mainly consider static factors such as complications, drugs, and the nature of surgery. This limitation makes these models not perform well enough for high-risk and elderly patients (11). Therefore, in the population of cardiac surgery and non-cardiac surgery, researchers are more willing to explore the impact of ambulatory blood pressure data on outcomes. It was found that there was a significant relationship between intraoperative blood pressure variability and postoperative adverse outcomes (4, 5, 12, 13). However, blood pressure variability is less effective in predicting death than traditional predictors (12). In outpatients with hypertension, studies have found that the daily average variability of family self-measured blood pressure for three consecutive days which is more than 11.0/12.8 is related to the occurrence of adverse cardiovascular outcomes. In addition, increased blood pressure variability in outpatients with hypertension can cause organ damage (3, 14). Similarly, in the outpatient population, some scholars pointed out that the variability indexes of family self-measured blood pressure and 24-hour ambulatory blood pressure can provide hints for organ injury (15). In critically ill patients, the researchers found that increased diurnal blood pressure variability (greater than 5%) or elevated nighttime blood pressure were associated with in-hospital mortality and long-term mortality (16, 17). At the same time, some studies have shown that the association between blood pressure variability and in-hospital mortality is weak in severe patients. However, the main endpoint of this study is acute renal injury during hospitalization, and the study population is not all severe patients (18). These studies have some defects. For example, the study population excludes the people who use vasoactive drugs and shock on the first day (17), the study factor variability index is divided into two categorical variables (16), or the study does not carry out nonlinear relationship tests (18).

As far as we know, our study is the first to study the relationship between blood pressure variability as a continuous variable and mortality by using the patient information of public database and to analyze the nonlinear relationship between them. The trend of the relationship between BPV and outcomes was similar to the outpatients with hypertension (3) and the patients undergoing surgery (19). As for ARV lower than 7.2 mmHg, lower BPV may be a risk factor. Some studies have shown that low heart rate variability is a marker of autonomic dysfunction (20, 21). Because physiological parameters such as blood pressure and heart rate are autonomic functions, decreased variability in blood pressure may be associated with increased mortality due to autonomic dysfunction as seen with heart rate. As for ARV higher than 7.2 mmHg, higher BPV was a risk factor. This result was consistent with the most of other BPV studies on patients with surgery (4, 5) or outpatients with hypertension (3). The higher or lower BPV may reflect the damage of cardiovascular homeostasis automatic regulation function and also cause potential pathological damage by affecting organ perfusion. That could explain our findings.

Our research has some potential limitations. First of all, this study is a retrospective design. Although we have adjusted many covariates and conducted subgroup analyses, some information such as the frequency of blood pressure monitoring, height of most patients, and so on are lost. Second, MIMIC-III is just a single-center database. Selection bias is inevitable. As the database contains different kinds of ICUs, including internal medicine, surgery, and emergency ICU, their data may reflect the real situation encountered by ICU doctors. Third, the blood pressure records in the database are not routine measurements, and there may be different time intervals between every two blood pressure records. This study only discussed the relationship between the blood pressure variability ARV and CV derived from hourly blood pressure records and mortality. In the future, a well-designed multi-center prospective study should be conducted to evaluate the causal relationship between blood pressure variability and mortality in critically ill patients with more accurate time-resolution blood pressure records.

## Conclusion

In critically ill patients, we identified a statistically significant association between BPV and in-hospital, 28-day, and 1-year mortality. This relationship is not a simple linear relationship, but a U-shaped curve relationship. Future studies should evaluate the utility of indices of BPV to understand if there is a clinically intervenable target to improve outcomes in critically ill patients.

## Data Availability Statement

The datasets presented in this study can be found in online repositories. The names of the repository/repositories and accession number(s) can be found below: https://physionet.org/content/mimiciii-demo/1.4/.

## Ethics Statement

The studies involving human participants were reviewed and approved by the Institutional Review Board of Beth Israel Deaconess Medical Center. Written informed consent for participation was not required for this study in accordance with the national legislation and the institutional requirements.

## Author Contributions

CH: conceptualization, methodology, software, data curation, and writing- reviewing and editing. XW: software and data curation. YL: methodology, software, and validation. FH: conceptualization and supervision. All authors contributed to the article and approved the submitted version.

## Conflict of Interest

XW was employed by Netbrain Technologies Inc. The remaining authors declare that the research was conducted in the absence of any commercial or financial relationships that could be construed as a potential conflict of interest.

## Publisher’s Note

All claims expressed in this article are solely those of the authors and do not necessarily represent those of their affiliated organizations, or those of the publisher, the editors and the reviewers. Any product that may be evaluated in this article, or claim that may be made by its manufacturer, is not guaranteed or endorsed by the publisher.

## Footnotes

1
http://www.R-project.org

## References

[1] Parati G Torlasco C Pengo M Bilo G Ochoa JE. Blood pressure variability: its relevance for cardiovascular homeostasis and cardiovascular diseases. *Hypertens Res.* (2020) 43:609–20. 10.1038/s41440-020-0421-5 32203448

[2] Stevens S Wood S Koshiaris C Law K Glasziou P McManus RJ Blood pressure variability and cardiovascular disease: systematic review and meta-analysis. *BMJ.* (2016) 354:i4098. 10.1136/bmj.i4098 PMC4979357 27511067

[3] Juhanoja E Niiranen T Johansson J Puukka PJ Thijs L Asayama K Outcome-driven thresholds for increased home blood pressure variability. *Hypertension.* (2017) 69:599–607. 10.1161/HYPERTENSIONAHA.116.08603 28193705

[4] Park S Lee H Jung C Choi Y Yoon H Kim S Intraoperative arterial pressure variability and postoperative acute kidney injury. *Clin J Am Soc Nephrol.* (2020) 15:35–46. 10.2215/CJN.06620619 31888922 PMC6946069

[5] Jinadasa SP Mueller A Prasad V Subramaniam K Heldt T Novack V Blood pressure coefficient of variation and its association with cardiac surgical outcomes. *Anesth Analg.* (2018) 127:832–9. 10.1213/ANE.0000000000003362 29624524

[6] Johnson AEW Pollard TJ Shen L Lehman LW Feng M Ghassemi M MIMIC-III, a freely accessible critical care database. *Sci Data.* (2016) 3:160035. 10.1038/sdata.2016.35 27219127 PMC4878278

[7] Johnson A Pollard T Mark R. *MIMIC-III Clinical Database Demo (version 1.4). PhysioNet*. Cambridge, MA: MIT Laboratory for Computational Physiology (2019). 10.13026/C2HM2Q

[8] Kikuya M Ohkubo T Metoki H Asayama K Hara A Obara T Day-by-day variability of blood pressure and heart rate at home as a novel predictor of prognosis: the Ohasama study. *Hypertension.* (2008) 52:1045–50. 10.1161/HYPERTENSIONAHA.107.104620 18981332

[9] Rothwell P Howard S Dolan E O’Brien E Dobson J Dahlof B Prognostic significance of visit-to-visit variability, maximum systolic blood pressure, and episodic hypertension. *Lancet.* (2010) 375:895–905. 10.1016/S0140-6736(10)60308-X 20226988

[10] Pierdomenico S Di Nicola M Esposito A Di Mascio R Ballone E Lapenna D Prognostic value of different indices of blood pressure variability in hypertensive patients. *Am J Hypertens.* (2009) 22:842–7. 10.1038/ajh.2009.103 19498342

[11] Leontyev S Walther T Borger M Lehmann S Funkat AK Rastan A Aortic valve replacement in octogenarians: utility of risk stratification with EuroSCORE. *Ann Thorac Surg.* (2009) 87:1440–5. 10.1016/j.athoracsur.2009.01.057 19379882

[12] Packiasabapathy S Prasad V Rangasamy V Popok D Xu X Novack V Cardiac surgical outcome prediction by blood pressure variability indices Poincaré plot and coefficient of variation: a retrospective study. *BMC Anesthesiol.* (2020) 20:56. 10.1186/s12871-020-00972-5 32126969 PMC7055104

[13] Aronson S Stafford-Smith M Phillips-Bute B Shaw A Gaca J Newman M. Intraoperative systolic blood pressure variability predicts 30-day mortality in aortocoronary bypass surgery patients. *Anesthesiology.* (2010) 113:305–12. 10.1097/ALN.0b013e3181e07ee9 20571360

[14] Manios E Tsagalis G Tsivgoulis G Barlas G Koroboki E Michas F Time rate of blood pressure variation is associated with impaired renal function in hypertensive patients. *J Hypertens.* (2009) 27:2244–8. 10.1097/HJH.0b013e328330a94f 19644388

[15] Wei F Li Y Zhang L Xu T Ding F Wang J Beat-to-beat, reading-to-reading, and day-to-day blood pressure variability in relation to organ damage in untreated Chinese. *Hypertension.* (2014) 63:790–6. 10.1161/HYPERTENSIONAHA.113.02681 24396027

[16] Gao Y Wang Q Li J Zhang J Li R Sun L Impact of mean arterial pressure fluctuation on mortality in critically Ill patients. *Crit Care Med.* (2018) 46:e1167–74. 10.1097/CCM.0000000000003435 30247271

[17] Li J Li R Gao Y Zhang J Zhao Y Zhang X Nocturnal mean arterial pressure rising is associated with mortality in the intensive care unit: a retrospective cohort study. *J Am Heart Assoc.* (2019) 8:e012388. 10.1161/JAHA.119.012388 31566067 PMC6806033

[18] Xie Z Liao X Yin W Kang Y Guo J Lu M. Relationship between short-term blood pressure variability and incidence of acute kidney injury in critically Ill patients. *Kidney Blood Press Res.* (2017) 42:1238–46. 10.1159/000485927 29248933

[19] Mascha E Yang D Weiss S Sessler D. Intraoperative mean arterial pressure variability and 30-day mortality in patients having noncardiac surgery. *Anesthesiology.* (2015) 123:79–91. 10.1097/ALN.0000000000000686 25929547

[20] Nolan J Batin P Andrews R Lindsay S Brooksby P Mullen M Prospective study of heart rate variability and mortality in chronic heart failure: results of the United Kingdom heart failure evaluation and assessment of risk trial (UK-heart). *Circulation.* (1998) 98:1510–6. 10.1161/01.cir.98.15.1510 9769304

[21] Tsuji H Larson M Venditti F Manders E Evans J Feldman C Impact of reduced heart rate variability on risk for cardiac events. The Framingham heart study. *Circulation.* (1996) 94:2850–5. 10.1161/01.cir.94.11.2850 8941112

